# Matrine induces Akt/mTOR signalling inhibition‐mediated autophagy and apoptosis in acute myeloid leukaemia cells

**DOI:** 10.1111/jcmm.13049

**Published:** 2016-12-27

**Authors:** Junqing Wu, Gang Hu, Yuqing Dong, Ruye Ma, Zhijie Yu, Songfu Jiang, Yixiang Han, Kang Yu, Shenghui Zhang

**Affiliations:** ^1^ Department of HematologyWenzhou Key Laboratory of HematologyThe First Affiliated Hospital of Wenzhou Medical UniversityWenzhouChina; ^2^ Laboratory of Internal MedicineThe First Affiliated Hospital of Wenzhou Medical UniversityWenzhouChina

**Keywords:** acute myeloid leukaemia, matrine, autophagy, apoptosis, Akt, mTOR

## Abstract

Pharmacological modulation of autophagy has been referred to as a promising therapeutic strategy for cancer. Matrine, a main alkaloid extracted from Sophora flavescens Ait, has antitumour activity against acute myelocytic leukaemia (AML). Whether autophagy is involved in antileukaemia activity of matrine remains unobvious. In this study, we demonstrated that matrine inhibited cell viability and colony formation *via* inducing apoptosis and autophagy in AML cell lines HL‐60, THP‐1 and C1498 as well as primary AML cells. Matrine promoted caspase‐3 and PARP cleavage dose‐dependently. Matrine up‐regulated the level of LC3‐II and down‐regulated the level of SQSTM1/p62 in a dose‐dependent way, indicating that autophagy should be implicated in anti‐AML effect of matrine. Furthermore, the autophagy inhibitor bafilomycin A1 relieved the cytotoxicity of matrine by blocking the autophagic flux, while the autophagy promoter rapamycin enhanced the cytotoxicity of matrine. Additionally, matrine inhibited the phosphorylation of Akt, mTOR and their downstream substrates p70S6K and 4EBP1, which led to the occurrence of autophagy. *In vivo* study demonstrated that autophagy was involved in antileukaemia effect of matrine in C57BL/6 mice bearing murine AML cell line C1498, and the survival curves showed that mice did benefit from treatment with matrine. Collectively, our findings indicate that matrine exerts antitumour effect through apoptosis and autophagy, and the latter one might be a potential therapeutic strategy for AML.

## Introduction

Acute myeloid leukaemia (AML) is a clonal haematopoietic disorder resulting from multiple genetic and epigenetic lesions affecting differentiation, proliferation and apoptosis 1. In particular, elevated expression of anti‐apoptotic molecules is a major mechanism of pathogenicity and chemotherapy resistance 2. Three main apoptotic pathways identified, including mitochondrial pathway, death receptor pathway and endoplasmic reticulum stress pathway, ultimately converge on the executive enzyme caspase‐3, and the activation of caspase‐3 leads to the activation of poly‐ADP‐ribose polymerase (PARP) 3.

Another type of cell death named autophagy is often confused with apoptosis. Autophagy is a self‐degradation process characterized by formation of double‐membrane vesicles called autophagosomes, which sequester excess or defective organelles and then fuse with lysosomes for degradation of enclosed materials 4. Multiple autophagic adapters, like SQSTM1/p62, are selectively degraded by autophagy and able to act as cargo receptors to directly interact with autophagosomal marker protein LC3 for efficient selective autophagy 5. Several signalling pathways, such as PI3K/Akt/mTOR/p70S6K pathway, have been demonstrated to be involved in the regulation of autophagy 6, 7, 8.

Generally, autophagy blocks the induction of apoptosis, and the activation of apoptosis‐associated molecules hinders the autophagic process 9. However, in some cases, autophagy degrades the cytoplasm excessively, leading to the autophagic cell death 10. In haematologic malignancies, autophagy, either acts as a chemoresistance mechanism or tumour‐suppressive function, might have broad applications to improve clinical outcome 11.

Matrine, a quinolizidine alkaloid that has been widely used to treat viral hepatitis, cardiac arrhythmia and skin inflammations, exhibits chemotherapeutic potential through its ability to trigger caspase‐independent program cell death 12. In our previous studies, we have found that matrine can be used in haematologic malignancies to induce cell apoptosis 13, 14. Although autophagy induction or inhibition has been reported to be implicated in the antitumour activity of matrine against several solid tumours 15, 16, 17, the role of autophagy and the underlying mechanism remain unobvious in AML.

In this study, we demonstrated that matrine solely induced both autophagy and apoptosis dose‐dependently in AML cells *in vitro*. After treatment in combination with rapamycin, apoptosis had been enhanced, while with bafilomycin A1 (Baf A1), the level of apoptosis decreased, suggesting that matrine‐induced autophagy promotes apoptosis. Finally, matrine alleviated tumour burden in a murine AML model, suggesting autophagy might play a crucial role.

## Materials and methods

### AML cell lines and primary AML cells

Human AML cell lines HL‐60 and THP‐1 (Cell Bank of Chinese Academy of Sciences, Shanghai, China) were cultured in RPMI 1640 containing 10% foetal bovine serum (FBS; Gibco, Grand Island, NY, USA). Murine AML cell line C1498 (Cell Bank of Chinese Academy of Sciences) was cultured in DMEM medium (Gibco) with 10% FBS. A total of 17 patients with newly diagnosed AML according to the World Health Organization classification system were enrolled in this study in our centre. Primary AML cells were isolated from bone marrow aspirates by Ficoll‐Hypaque Solution (Haoyang Institute of Biotechnology, Tianjin, China) and then cultured in RPMI 1640 containing 15% FBS. This study was approved by the Institutional Review Board of the First Affiliated Hospital of Wenzhou Medical University, and informed consent was obtained from all participants in accordance with the Declaration of Helsinki protocol. All the cells were grown at 37°C in a 5% CO_2_ atmosphere incubator.

### Cell viability assay

AML cell lines HL‐60, THP‐1 and C1498 as well as primary AML cells were seeded in 96‐well plates at a density of 8 × 10^3^ cells/well and treated with various doses of matrine (Tianyuan Biological Agent Plant, Xi'an, Shanxi, China) with or without 10 nM Baf A1 (Meilun Biological Agent Plant, Dalian, Liaoning, China) or 20 nM rapamycin (Selleck Chemicals, Houston, TX, USA) or 10 μM z‐VAD‐FMK (Selleck Chemicals). After 12, 24 or 48 hrs, cell viability was measured using the Cell Counting Kit‐8 (CCK‐8) assay (Dojindo, Kumamoto, Japan) according to the manufacturer's instruction. The absorbance was measured at 450 nm using an ELISA reader (Elx 800; Bio‐Tek Instruments, Winooski, VT, USA).

### Colony formation assay

AML cells were diluted and seeded at about 1 × 10^4^ cells/well in 35‐mm dishes with or without matrine (1.5 g/l) in methylcellulose medium (MethoCult™ H4034, Stemcell Technologies, Vancouver, BC, Canada). After incubation for 8 days in a 5% CO_2_ atmosphere incubator at 37°C, the cells were examined using an inverted microscope (Olympus, Tokyo, Japan) equipped with a CCD camera. The colony is defined to consist of at least 40 cells, and visible colonies were counted. Cells were then washed with PBS twice and counted using haemocytometer.

### Western blot assay

Western blot analysis was performed as previously described 18. Briefly, after treatment with different concentrations of matrine, the cells were collected and lysed immediately using RIPA Lysis Buffer (Beyotime Institute of Biotechnology, Haimen, China) containing PMSF and Halt protease and phosphatase inhibitor cocktail (Pierce, Rockford, IL, USA). Protein concentration was measured using BCA protein assay kit (Beyotime Institute of Biotechnology). The protein was boiled and subjected to Western blot with antibodies against LC3 II, SQSTM1/p62, PARP, caspase‐3, phospho‐Akt (Ser473), Akt, phospho‐mTOR (Ser2448), mTOR, phospho‐p70S6K (Thr389), p70S6K, phospho‐4EBP1 (Thr37/46), 4EBP1 or β‐actin (all from Cell Signaling Technology, Beverly, MA, USA), respectively. Optical densities of the bands were analysed with Bio‐Rad image analysis (Bio‐Rad, Hercules, CA, USA).

### Apoptosis assay and cell cycle assay

AML cells were exposed to various doses of matrine with or without Baf A1 and rapamycin for 24 hrs in 24‐well plates at a density of 2 × 10^5^ cells/well. Then, apoptosis was determined using FITC Annexin Apoptosis Detection Kit II (BD Biosciences, San Diego, CA, USA), and cell cycle analysis was performed using Cell Cycle Staining Kit (MultiSciences, Hangzhou, China) on a flow cytometry (FACSCalibur; BD Biosciences) according to the manufacturer's protocols.

### *In vivo* studies

C57BL/6 mice (6–8 weeks old/20–25 g bodyweight) were purchased from Laboratory Animal Centre of Wenzhou Medical University. Exponentially growing C1498 cells (1 × 10^7^) were suspended in 100 μl PBS, and then subcutaneously injected into the tail vein of recipient mice, which had been already exposed to 4 Gy myeloablative irradiation 4 hrs before. On day 7, mice were randomly divided into four groups, with 15 animals each group. The treatment groups were injected intraperitoneally with matrine (50 mg/kg) or chloroquine (30 mg/kg; Sigma‐Aldrich, St. Louis, MO, USA) or both drugs on alternative days, respectively, while the vehicle group was given saline. Five mice from each group were killed on day 28. The spleen and femur were dissected out for immunohistochemistry (IHC) analysis. The spleen was weighed by an electronic balance (MS105DU; Mettler Toledo, Bradford, MA, USA). Bone marrow mononuclear cells were isolated from femur and tibia of C57BL/6 mice and lysed immediately for Western blot analysis. The peripheral blood and bone marrow smears were air‐dried and stained with Wright's stain, and the immature leucocytes were counted under a microscope (BX51; Olympus). Remaining 10 mice from each group were observed 50 days for survival rates. Animal procedures were carried out in accordance with institutional guidelines after Whenzhou Medical University Animal Care and Use Committee approved the study protocol.

### Immunohistochemistry analysis

Spleen and femur bones were dissected out and fixed with 10% paraformaldehyde and embedded in paraffin. Sections were deparaffinized and incubated with antibodies against LC3 II and SQSTM1/p62 (Cell Signaling Technology) followed by visualization with the one‐step polymer detection system (ZSGB‐bio company, Beijing, China). To visualize the expression of SQSTM1/p62 and LC3 II, images of bone and spleen were captured using a microscope with CCD. To quantify the level of SQSTM1/p62 and LC3 II, the integrated optical density (IOD) of different regions of the spleen and bone was measured automatically using Image‐Pro Plus software (Media Cybernetics, Silver Spring, MD, USA).

### Statistical analysis

Data presented as mean ± S.E.M. were representative of at least three independent experiments. Statistical analyses were performed by one‐way analysis of variance (anova). *P* values less than 0.05 were considered statistically significant.

## Results

### Matrine induces growth inhibition in AML cells

**Figure 1:**
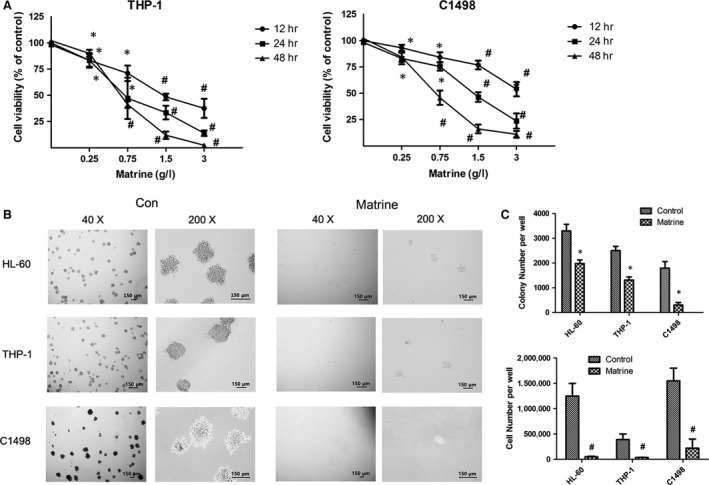
Matrine induces growth inhibition in AML cells. (**A**) After incubated with various concentration of matrine for 12, 24 and 48 hrs, the cell viability of THP‐1 and C1498 cells was measured by CCK‐8 assay. (**B**) The morphological changes of colonies were observed under a microscopy in AML cell lines HL‐60, THP‐1 and C1498 with or without 1.5 g/l matrine for 8 days. Representative images were shown. (**C**) Quantitative data of colony and cell number were presented in bar charts. Results were expressed as mean ± S.E.M. representing at least three independent experiments. *P <* *0.05, #*P < *0.01, *versus* the respective control.

Firstly, the short‐term inhibitory effect of matrine was determined on AML cell lines HL‐60, THP‐1, C1498 and primary AML cells using CCK‐8 assay. We had demonstrated that matrine significantly reduced the cell viabilities of HL‐60 cells and primary AML cells in a time‐ and dose‐dependent manner in our previous study 13, and this phenomenon was also found on THP‐1 and C1498 cells (Fig. 1A). Furthermore, we measured long‐term growth inhibitory effect using colony formation assay. After 8 days of incubation, matrine at 1.5 g/l markedly inhibited colony formation characterized by small colony size in AML cell lines HL‐60, THP‐1 and C1498 (Fig. 1B and C). Both colony number and total cell number were significantly reduced by matrine in all three AML cell lines tested (Fig. 1C). Meanwhile, we used daunorubicin, a classic chemotherapeutic drug for AML, as a positive control, and surprisingly, the effect of matrine on AML cells was more obvious than that of 100 nM daunorubicin (Fig. S1). These data further confirmed that matrine potently inhibits cell growth and colony formation in AML cells.

### Matrine induces autophagy in AML cells

**Figure 2:**
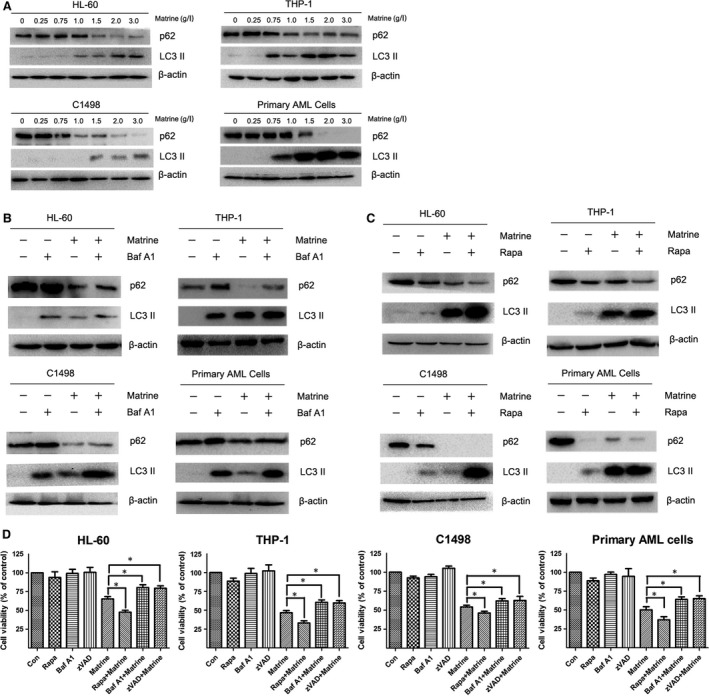
Matrine induces autophagy in AML cells. (**A**) HL‐60, THP‐1, C1498 and primary AML cells were treated with 0, 0.25, 0.75, 1, 1.5, 2, 3 g/l matrine for 24 hrs, and the autophagy markers LC3 II and SQSTM1/p62 were detected by Western blot analysis. (**B ‐ C**) AML cells were treated with 1.5 g/l matrine in the presence or absence of 10 nM bafilomycin A1 (Baf A1) or 20 nM rapamycin (Rapa) for 24 hrs, and the levels of LC3 II and SQSTM1/p62 were assessed by Western blot analysis. (**D**) After treatment with 1.5 g/l matrine in the presence or absence of 10 nM Baf A1 or 20 nM rapamycin or 10 μM z‐VAD‐FMK (zVAD) for 24 hrs, the cell viability was measured by CCK‐8 assay. Results were expressed as mean ± S.E.M. representing at least three independent experiments. *P <* *0.05, *versus* matrine alone group.

Matrine has been demonstrated to induce apoptosis in AML cells 13. The fact that apoptosis often occurs simultaneously with autophagy in the same cell interested us to investigate whether autophagy is also involved in anti‐AML effect of matrine. Firstly, we investigated the levels of SQSTM1/p62 and LC3 II, which are two key proteins in autophagy regulation after treatment with matrine for 24 hrs. As shown in Figure 2A, the accumulation of LC3 II and down‐regulation of SQSTM1/p62 were observed in AML cells, indicating that matrine induced autophagic flux in a dose‐dependent manner. To further confirm whether matrine induces autophagy in AML cells, an autophagy inhibitor Baf A1, the lysosomotropic agent that inhibits lysosomal degradation of autophagosome, was employed. Inhibition of autophagy at an early stage results in decreased production of LC3 II, but inhibition of autophagic flux at a late stage using Baf A1 or chloroquine leads to increased levels of LC3 II 19. As expected, when combined with Baf A1, the matrine‐induced attenuation of SQSTM1/p62 was partly reversed with the level of LC3 II still gradually increasing, revealing that Baf A1 delayed the matrine‐induced autophagy (Fig. 2B). In contrast, an autophagy inducer rapamycin promoted matrine‐induced autophagy as the level of SQSTM1/p62 decreased, whereas the level of LC3 II increased (Fig. 2C). Thus, autophagy indeed occurred in matrine‐treated AML cells. Subsequently, we investigated the role of autophagy in anti‐AML effects of matrine. As shown in Figure 2D, Baf A1 blocked the cell growth inhibition induced by matrine, while rapamycin cooperated with matrine to reduce cell viability at 24 hrs after exposure. However, Baf A1 almost did not influence the colony formation of HL‐60 cells treated by matrine (Fig. S2). These findings strongly suggest that autophagy is induced in AML cells after matrine treatment.

### Matrine induces caspase‐dependent apoptosis, following cell cycle arrest

**Figure 3:**
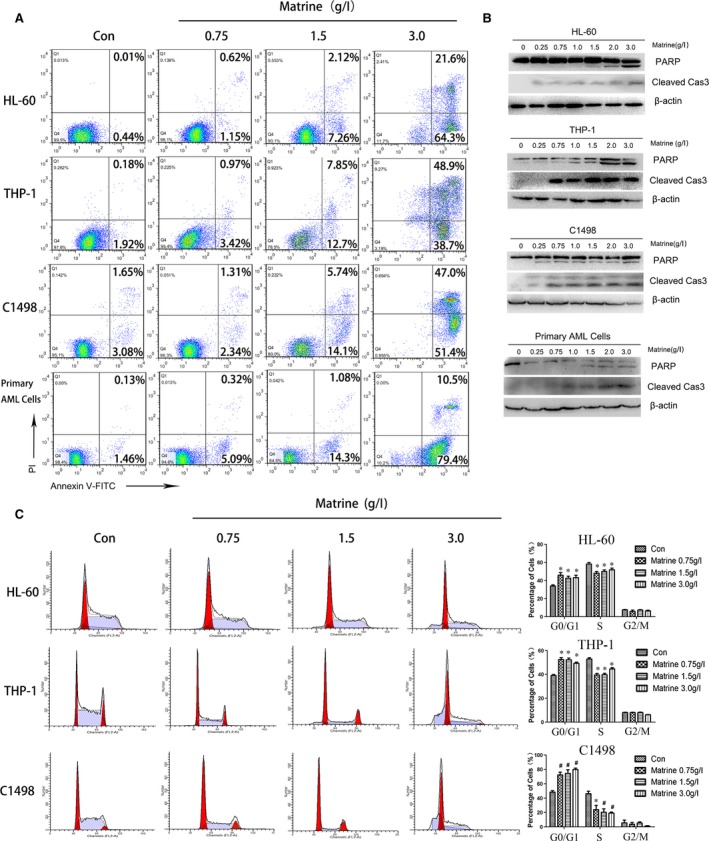
Matrine induces caspase‐3‐dependent apoptosis, following cell cycle arrest. (**A**) AML cells were treated with 0, 0.75, 1.5, 3 g/l matrine for 24 hrs and stained with Annexin V/PI before being analysed by flow cytometry. (**B**) AML cells were treated with various concentrations of matrine, and Western blot analysis was performed to assess the cleavage level of PARP and caspase‐3. (**C**) The cell cycle was analysed using PI staining by flow cytometry in AML cells treated with 0, 0.75, 1.5, 3 g/l matrine for 24 hrs, and the percentage of cells in different phases was presented in bar charts. Results were expressed as mean ± S.E.M. representing at least three independent experiments. *P <* *0.05, #*P < *0.01, *versus* the respective control.

We next determined whether matrine was cytostatic or cytotoxic against AML cells. As shown in Figure 3A, matrine treatment dramatically promoted apoptosis in a dose‐dependent manner, especially above 1.5 g/l. And the cleavages of caspase‐3 and PARP were determined to further assess apoptosis. As shown in Figure 3B, cleaved caspase‐3 and cleaved PARP both increased as the concentration levels rose, especially above 1.5 g/l. z‐VAD‐FMK, a pan‐caspase inhibitor, was also employed to evaluate whether apoptosis alone determined the cell fate. When only treated for 24 hrs, z‐VAD‐FMK moderately reduced the cytotoxicity of matrine against AML cells (Fig. 2D). However, at 8 days after cotreatment with matrine using the colony formation assay, z‐VAD‐FMK made little effort to rescue the matrine‐treated HL‐60 cells (Fig. S2). Additionally, cell cycle distribution was performed and the results showed that matrine led to an increase in G1 phase with diminished S phase in AML cells, suggesting that matrine is able to induce G1 arrest to decelerate the cell cycle and prevent the cells from entering the S phase and proliferating (Fig. 3C). These results reveal that besides autophagy activation, matrine also causes apoptosis in a dose‐dependent way.

### Autophagy promoted by matrine potentiates apoptotic effect in AML cells

**Figure 4:**
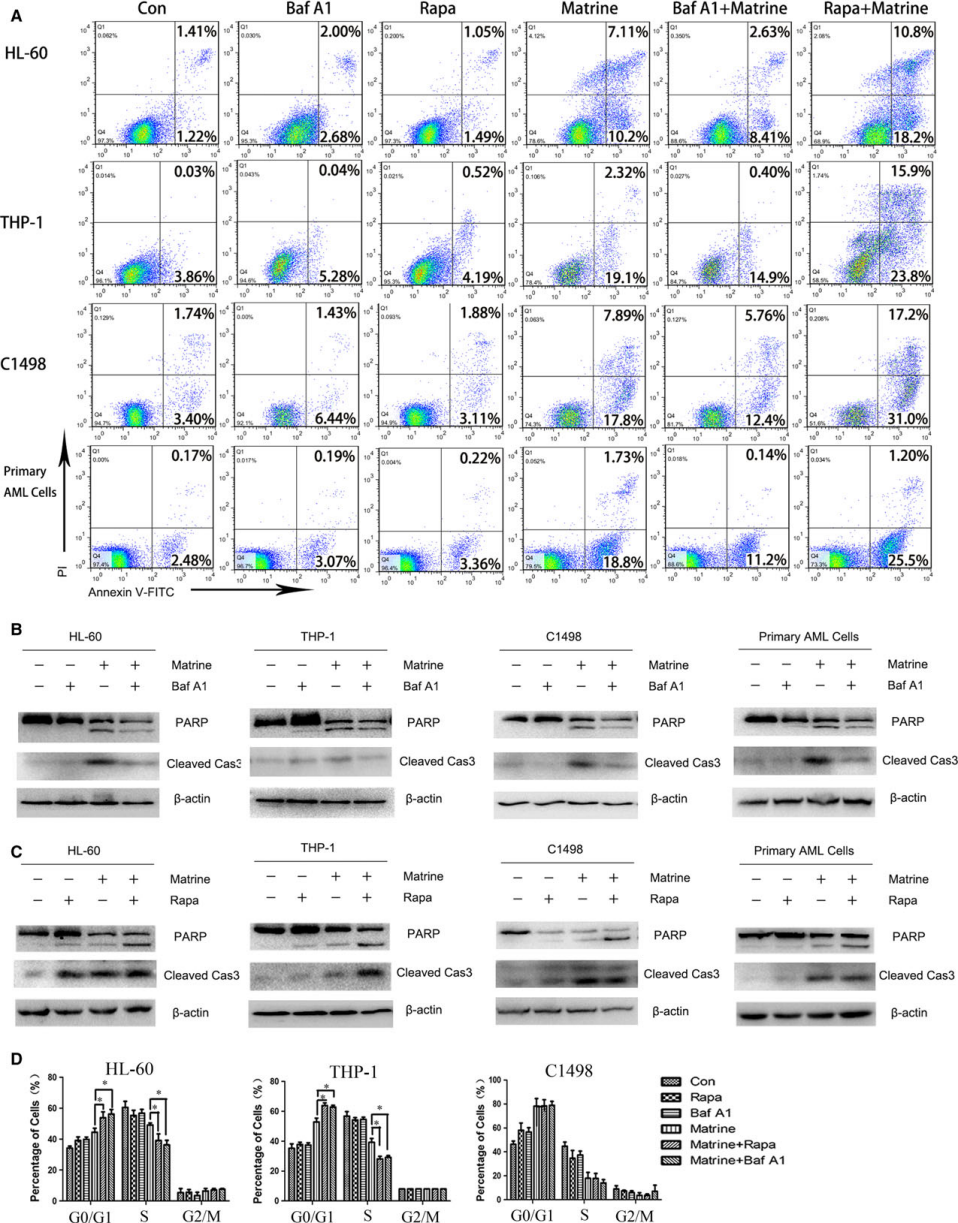
Autophagy promoted by matrine potentiates apoptotic effect in AML cells. AML cells were treated with 1.5 g/l matrine, either alone or in combination with 10 nM Baf A1 or 20 nM rapamycin (Rapa) for 24 hrs, and subsequently stained with Annexin V/PI for flow cytometry analysis (**A**) or determined for the cleavage of PARP and caspase‐3 using Western blot analysis (**B ‐ C**), or stained with PI to analyse the cell cycle using flow cytometry, and the percentage of cells in different phases was presented in bar charts (**D**). Results were expressed as mean ± S.E.M. representing at least three independent experiments. *P <* *0.05, *versus* matrine alone group.

Once proved, autophagy and apoptosis were both involved in matrine‐treated AML cells, the underlying relationship aroused our curiosity. First of all, we tested the apoptosis rate when the autophagy was inhibited or induced. As described before, Baf A1 and rapamycin were employed. Apoptosis assay manifested that Baf A1 or rapamycin alone did not induce apoptosis in AML cells. Baf A1 potently inhibited matrine‐induced apoptosis, while rapamycin dramatically promoted matrine‐induced apoptosis (Fig. 4A and Fig. S3). Furthermore, the levels of cleaved caspase‐3 and PARP under the same condition were analysed. After co‐incubation with Baf A1, matrine‐induced cleavages of caspase‐3 and PARP were both reduced (Fig. 4B). Rapamycin treatment alone slightly increased the levels of cleaved caspase‐3 and cleaved PARP. However, cotreatment with rapamycin and matrine for 24 hrs, all AML cells exhibited an evidently higher level of cleaved caspase‐3 and PARP (Fig. 4C). Finally, cell cycle distribution illustrated that rapamycin exaggerated the ability of matrine to arrest AML cells at G1 phase and stopped them from entering S phase. Surprisingly, the effect of Baf A1 at G1 and S phase was similar to rapamycin (Fig. 4D). These data reveal that matrine and rapamycin exert synergistic effect to enhance apoptosis, and matrine combined with Baf A1 has an opposite effect.

### Akt/mTOR signalling pathway is involved in matrine‐induced autophagy in AML cells

**Figure 5:**
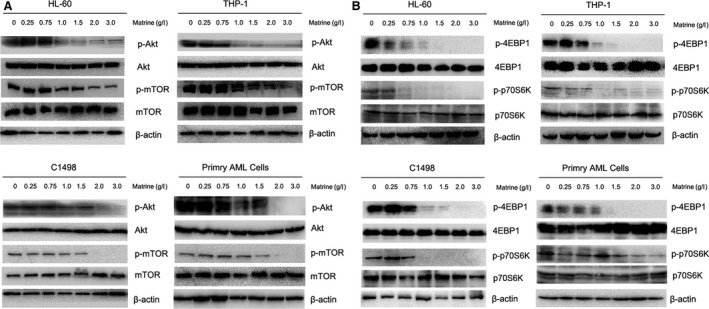
AKT/mTOR signalling pathway is involved in matrine‐induced autophagy in AML cells. After treated with various concentrations of matrine for 24 hrs, the levels of (**A**) p‐Akt, Akt, p‐mTOR, mTOR, (**B**) p70S6K, p‐p70S6K, 4EBP1 and p‐4EBP1 were analysed by Western blot. Images shown were representatives of at least three independent experiments.

Akt/mTOR signalling pathway is involved in various physiological processes, including metabolism, cell growth, proliferation, differentiation, apoptosis and autophagy 20. To confirm whether the Akt/mTOR signalling pathway is related to matrine‐induced autophagy, we evaluated the level of phosphorylated Akt, mTOR and two downstream substrates named p70S6K and 4EBP1. As shown in Figure 5A and B, matrine significantly inhibited the phosphorylation of Akt, mTOR, p70S6K and 4EBP1 in a dose‐dependent manner in AML cell lines HL‐60, THP‐1 and C1498 as well as primary AML cells. These findings manifest that matrine negatively regulates the phosphorylation of Akt/mTOR signalling pathway to promote autophagy.

### Matrine‐induced autophagy alleviates AML burden *in vivo*

**Figure 6:**
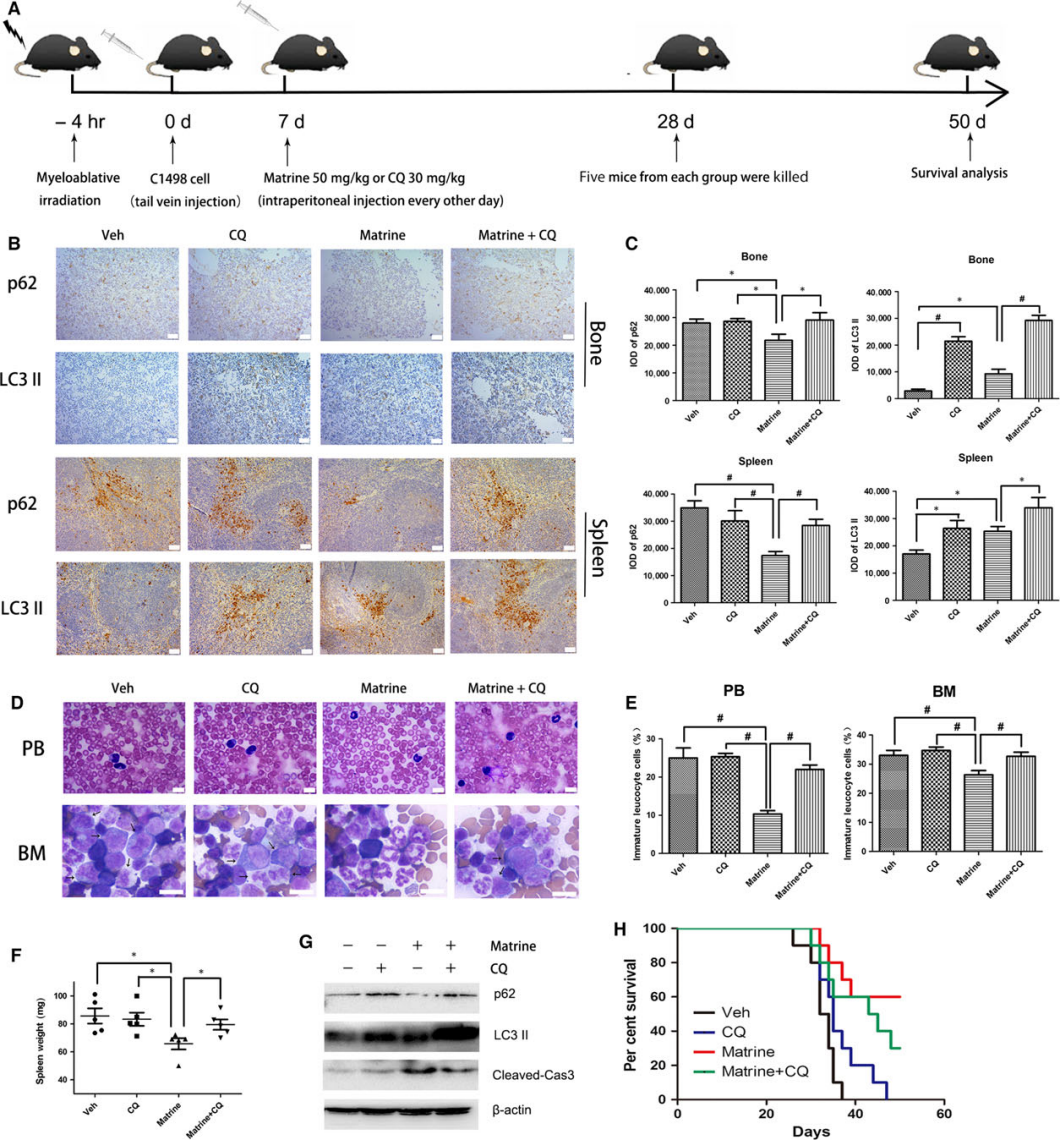
Autophagy promotion is involved in the anti‐AML effect of matrine *in vivo*. (**A**) Experimental protocol was used to assess the anti‐AML effect of matrine on C57BL/6 mice. (**B**) After treated with matrine, chloroquine (CQ) or saline (Veh), IHC staining of SQSTM1/P62 and LC3 II in spleen and bone were observed by microscopy and representative images were showed. The scale bars are 50 μm. (**C**) IOD of SQSTM1/P62 and LC3 II of spleen and bone were showed in bar charts. (**D**) Wright's staining of peripheral blood and bone marrow was performed to observe immature leucocyte cells by microscopy, and representative images are showed. The scale bars are 10 μm, and arrows indicate immature leucocyte cells. (**E**) The percentage of immature leucocyte cells in peripheral blood and bone marrow was shown. (**F**) The spleen weight of mice was evaluated after killed. (**G**) The levels of SQSTM1/p62, LC3 II and cleaved caspase‐3 in bone marrow mononuclear cells were determined using Western blot analysis. (**H**) Kaplan–Meier curves for survival were assessed for 10 mice per group, and the long‐rank test was used to evaluate the statistical differences in survival. Results were expressed as mean ± S.E.M., and images shown were representatives of at least three independent experiments. *P <* *0.05, #*P < *0.01, *versus* matrine alone group.

We established an AML model in C57BL/6 mice to investigate the role of autophagy *in vivo*. After myeloablative irradiation, C1498 cells were injected *via* tail vein, following intraperitoneal administration of matrine alone or with chloroquine every other day (Fig. 6A). Chloroquine, similarly to Baf A1, is an autophagy inhibitor blocking autophagy flux at late stage. The levels of SQSTM1/p62 and LC3 II in bone and spleen were determined by IHC,and the results indicated autophagy was promoted in matrine group, and blocked by chloroquine (Fig. 6B and C). Wright's staining showed that the percentage of immature leucocyte cells decreased in matrine group, both in peripheral blood and bone marrow compared with chloroquine group, suggesting that matrine‐reduced AML tumour burden is associated with autophagy induction (Fig. 6D and E). A reduction in spleen weight was also found in matrine‐treated group (Fig. 6F), further indicating the AML infiltration was alleviated. Matrine down‐regulated the level of SQSTM1/p62, accompanied by the up‐regulation of LC3 II and cleaved caspase‐3 in bone marrow mononuclear cells in matrine group (Fig. 6G). Kaplan–Meier survival curves showed that treatment of mice with matrine significantly decreased the mortality rate compared with mice that received vehicle (*P *<* *0.01; Fig. 6H). Chloroquine treatment alone did not significantly affect the survival rates of mice challenged with C1498 cells. In combination with matrine, chloroquine had a slight, but not significant, effect on the survival time of mice challenged with C1498 cells compared with mice that received matrine alone (*P *=* *0.053; Fig. 6H). These results indicated that the elevated autophagy level is at least partially related to the antileukaemic effect of matrine *in vivo*, which might be another highlight on AML therapy.

## Discussion

As a neoplastic blood disease with a 5‐year overall survival of only 3 to 8% in patients over 60 years old, the prognosis of AML remains dismal 21. Recent studies have indicated that regulators of apoptosis are recognized as novel therapeutic targets in AML 22, 23. A large group of natural compounds are proved to exert anticancer activity through apoptosis pathway 24. Multiple studies have demonstrated that matrine can induce apoptosis against many solid tumours and haematological malignancies 12, 13, 14.

Besides apoptosis, or type I programmed cell death (PCD), whether matrine‐induced autophagy, or type II PCD, jointly decides the fate of AML cells remains unobvious 25. To date, the precise role of autophagy in AML and whether matrine inhibits or promotes autophagy are controversial 16, 17, 26, 27. In our present study, matrine induced autophagy, characterized by up‐regulation of LC3 II and down‐regulation of SQSTM1/p62 in AML cells. Autophagy inhibitor Baf A1 and autophagy inducer rapamycin were used to further confirm the existence of autophagy induced by matrine. Matrine‐induced autophagy exerted a cytotoxicity rather than cytoprotection effect as rapamycin accelerated while Baf A1 relieved the inhibitory effect of matrine. In general, matrine‐induced autophagy increases cell death of AML cells.

The relationship between autophagy and apoptosis is complex 28. In some circumstances, autophagy potentiates the apoptotic effect in AML 29, but others diametrically opposed 30. The underlying mechanisms refer to cell types, phase, genetic background and microenvironment 31. In the present study, AML cells treated by matrine underwent cell cycle arrest and consequently succumbed to apoptosis when the cleavage levels of caspase‐3 and PARP increased in a dose‐dependent way. Further, Baf A1 decreased while rapamycin increased apoptosis in matrine‐treated AML cells. Additionally, we noticed that Baf A1 rescued matrine‐treated AML cells from apoptosis, whereas it still blocked the cell cycle in G1 phase. Baf A1 has been reported to induce apoptosis followed by cell cycle arrest 32, 33. However, our data suggested that even AML cells survive from low‐dose Baf A1, the retardation effect has already began to affect the cell cycle.

The blockage of Akt/mTOR signalling pathway plays a critical role in regulating autophagy 34, 35. Targeting mTOR has been proved to be effective for leukaemia therapy 36. The inactivation of the phosphorylation of Akt, mTOR and their downstream substances, p70S6K and 4EBP1, were found in matrine‐treated AML cells. Thus, matrine‐related autophagy might potentially reduce the drug resistance in AML.

To understand the functional role of matrine in the regulation of autophagy *in vivo*, we carried it out on C57BL/6 leukaemia mice *via* intraperitoneal injection of matrine. Although several studies have shown that matrine inhibits the growth of multiple tumour cells *in vivo* 12, 13, 37, the role of autophagy remains unobvious in matrine‐treated haematological tumour. In the present study, chloroquine, an autophagic inhibitor preventing autophagosomal fusion with the lysosome 38, was introduced to determine the autophagic role of matrine. The results of LC3 II and SQSTM1/p62 in Western blot analysis and IHC analysis revealed that matrine promoted intra‐ and extramedullary autophagy, along with apoptosis. Furthermore, we found the percentage of immature leucocyte cells in peripheral blood and bone marrow decreased in matrine‐treated group, indicating that the tumour burden is reduced. Moreover, the losses of weight in matrine‐treated leukaemic spleen further verified that matrine could alleviate the infiltration. Matrine significantly prolonged the survival time in the murine model transplanted with C1498 cells. There was not a significant difference between chloroquine plus matrine group and matrine alone group during the 50‐day observation period. We think that chloroquine treatment can shorten the survival time of mice treated with matrine as the observation time increases. Therefore, the effects reversed by the addition of chloroquine suggested that promoting autophagy did make sense in cancer therapy in AML.

In conclusion, our present study has demonstrated that matrine induces autophagy and apoptosis in AML *in vitro* and *in vivo*, and matrine‐induced apoptosis partially results from autophagy. Additionally, Akt/mTOR pathway is involved in autophagy of matrine‐treated AML cells. It is highlighted that the autophagic promotion of matrine might be a promising therapeutic strategy for AML.

## Conflict of interest statement

The authors declare that they have no conflict of interest.

## Supporting information


**Figure S1** After incubated with matrine (1.5 g/l) or daunorubicin (100 nM), the cell viability of AML cell lines HL‐60, THP‐1, C1498 and primary AML cells was measured by CCK‐8 assay. *P <* *0.05, #*P *<* *0.01, *versus* the respective control.

**Figure S2** (A) The morphological changes of colonies were observed under a microscopy in HL‐60 cells treated with 1.5 g/l matrine, 10 nM Baf A1, or 10 μM z‐VAD‐FMK for 8 days. Images shown were representatives of at least three independent experiments. (B) Quantitative data of colony and cell number were presented in bar charts. Results were expressed as mean ± S.E.M. representing at least three independent experiments. *P <* *0.05, #*P *<* *0.01, *versus* the respective control.

**Figure S3** AML cell lines HL‐60, THP‐1, and C1498 as well as primary AML cells were treated with 1.5 g/l matrine, either alone or in combination with 10 nM Baf A1 or 20 nM rapamycin (Rapa) for 24 hrs, and subsequently stained with Annexin V/PI for flow cytometry analysis. The percentage of apoptosis was presented in bar charts. Results were expressed as mean ± S.E.M. representing at least three independent experiments. *P <* *0.05, *versus* matrine alone group.

**Table S1** Characteristics of patients with acute myeloid leukemia.

## References

[1] Dohner H , Weisdorf DJ , Bloomfield CD . Acute Myeloid Leukemia. N Engl J Med. 2015; 373: 1136–52. 26376137 10.1056/NEJMra1406184

[2] Khan I , Altman JK , Licht JD . New strategies in acute myeloid leukemia: redefining prognostic markers to guide therapy. Clin Cancer Res. 2012; 18: 5163–71. 22893630 10.1158/1078-0432.CCR-12-0313 PMC3902112

[3] Zeng W , Wang X , Xu P , *et al* Molecular imaging of apoptosis: from micro to macro. Theranostics. 2015; 5: 559–82. 25825597 10.7150/thno.11548 PMC4377726

[4] Boya P , Reggiori F , Codogno P . Emerging regulation and functions of autophagy. Nat Cell Biol. 2013; 15: 713–20. 23817233 10.1038/ncb2788 PMC7097732

[5] Johansen T , Lamark T . Selective autophagy mediated by autophagic adapter proteins. Autophagy. 2011; 7: 279–96. 21189453 10.4161/auto.7.3.14487 PMC3060413

[6] Saiki S , Sasazawa Y , Imamichi Y , *et al* Caffeine induces apoptosis by enhancement of autophagy *via* PI3K/Akt/mTOR/p70S6K inhibition. Autophagy. 2011; 7: 176–87. 21081844 10.4161/auto.7.2.14074 PMC3039768

[7] Fan G , Yu J , Asare PF , *et al* Danshensu alleviates cardiac ischaemia/reperfusion injury by inhibiting autophagy and apoptosis *via* activation of mTOR signalling. J Cell Mol Med. 2016; 20: 1908–19. 27385290 10.1111/jcmm.12883 PMC5020629

[8] Li J , Zhou J , Zhang D , *et al* Bone marrow‐derived mesenchymal stem cells enhance autophagy *via* PI3K/AKT signalling to reduce the severity of ischaemia/reperfusion‐induced lung injury. J Cell Mol Med. 2015; 19: 2341–51. 26177266 10.1111/jcmm.12638 PMC4594676

[9] Dong Z , Liang S , Hu J , *et al* Autophagy as a target for hematological malignancy therapy. Blood Rev. 2016; 30: 369–80. 27132115 10.1016/j.blre.2016.04.005

[10] Marino G , Niso‐Santano M , Baehrecke EH , *et al* Self‐consumption: the interplay of autophagy and apoptosis. Nat Rev Mol Cell Biol. 2014; 15: 81–94. 24401948 10.1038/nrm3735 PMC3970201

[11] Nencioni A , Cea M , Montecucco F , *et al* Autophagy in blood cancers: biological role and therapeutic implications. Haematologica. 2013; 98: 1335–43. 24006406 10.3324/haematol.2012.079061 PMC3762088

[12] Zhou H , Xu M , Gao Y , *et al* Matrine induces caspase‐independent program cell death in hepatocellular carcinoma through bid‐mediated nuclear translocation of apoptosis inducing factor. Mol Cancer. 2014; 13: 59. 24628719 10.1186/1476-4598-13-59 PMC4007561

[13] Zhang S , Zhang Y , Zhuang Y , *et al* Matrine induces apoptosis in human acute myeloid leukemia cells *via* the mitochondrial pathway and Akt inactivation. PLoS One. 2012; 7: e46853. 23056487 10.1371/journal.pone.0046853 PMC3466205

[14] Han Y , Zhang S , Wu J , *et al* Matrine induces apoptosis of human multiple myeloma cells *via* activation of the mitochondrial pathway. Leuk Lymphoma. 2010; 51: 1337–46. 20528251 10.3109/10428194.2010.488708

[15] Vakifahmetoglu‐Norberg H , **Xia** H‐G , Yuan J Pharmacologic agents targeting autophagy. J Clin Invest. 2015; 125: 5–13. 25654545 10.1172/JCI73937 PMC4382252

[16] Li Y , Zhang J , Ma H , *et al* Protective role of autophagy in matrine‐induced gastric cancer cell death. Int J Oncol. 2013; 42: 1417–26. 23404079 10.3892/ijo.2013.1817

[17] Zhang JQ , Li YM , Liu T , *et al* Antitumor effect of matrine in human hepatoma G2 cells by inducing apoptosis and autophagy. World J Gastroenterol. 2010; 16: 4281–90. 20818811 10.3748/wjg.v16.i34.4281 PMC2937108

[18] Qian S , Sun L , Li J , *et al* MAP30 inhibits autophagy through enhancing acetyltransferase p300 and induces apoptosis in acute myeloid leukemia cells. Oncol Rep. 2016; 35: 3705–13. 27035419 10.3892/or.2016.4705

[19] Mizushima N , Yoshimori T , Levine B . Methods in mammalian autophagy research. Cell. 2010; 140: 313–26. 20144757 10.1016/j.cell.2010.01.028 PMC2852113

[20] Cho CH . Frontier of epilepsy research ‐ mTOR signaling pathway. Exp Mol Med. 2011; 43: 231–74. 21467839 10.3858/emm.2011.43.5.032 PMC3104248

[21] Pandolfi A , Stanley RF , Yu Y , *et al* PAK1 is a therapeutic target in acute myeloid leukemia and myelodysplastic syndrome. Blood. 2016; 126: 1118–27. 10.1182/blood-2014-12-618801 PMC4551362 26170031

[22] Carter BZ , Qiu YH , Zhang N , *et al* Expression of ARC (apoptosis repressor with caspase recruitment domain), an antiapoptotic protein, is strongly prognostic in AML. Blood. 2016; 117: 780–7. 10.1182/blood-2010-04-280503 PMC3035072 21041716

[23] Hajj HE , Dassouki Z , Berthier C , *et al* Retinoic acid and arsenic trioxide trigger degradation of mutated NPM‐1 resulting in apoptosis of AML cells. Blood. 2015; 125: 3447–54. 25800051 10.1182/blood-2014-11-612416

[24] Millimouno FM , Dong J , Yang L , *et al* Targeting apoptosis pathways in cancer and perspectives with natural compounds from mother nature. Cancer Prev Res (Phila). 2014; 7: 1081–107. 25161295 10.1158/1940-6207.CAPR-14-0136

[25] Ouyang L , Shi Z , Zhao S , *et al* Programmed cell death pathways in cancer: a review of apoptosis, autophagy and programmed necrosis. Cell Prolif. 2012; 45: 487–98. 23030059 10.1111/j.1365-2184.2012.00845.x PMC6496669

[26] Altman JK , Szilard A , Goussetis DJ , *et al* Autophagy is a survival mechanism of acute myelogenous leukemia precursors during dual mTORC2/mTORC1 targeting. Clin Cancer Res. 2014; 20: 2400–9. 24610825 10.1158/1078-0432.CCR-13-3218 PMC4056773

[27] Fang J , Rhyasen G , Bolanos L , *et al* Cytotoxic effects of bortezomib in myelodysplastic syndrome/acute myeloid leukemia depend on autophagy‐mediated lysosomal degradation of TRAF6 and repression of PSMA1. Blood. 2012; 120: 858–67. 22685174 10.1182/blood-2012-02-407999 PMC3412348

[28] Maycotte P , Thorburn A . Autophagy and cancer therapy. Cancer Biol Ther. 2011; 11: 127–37. 21178393 10.4161/cbt.11.2.14627 PMC3047083

[29] Torgersen ML , Engedal N , Boe SO , *et al* Targeting autophagy potentiates the apoptotic effect of histone deacetylase inhibitors in t(8;21) AML cells. Blood. 2013; 122: 2467–76. 23970379 10.1182/blood-2013-05-500629

[30] Qin L , Tian Y , Zhang C , *et al* Targeting PDK1 with dichloroacetophenone to inhibit acute myeloid leukemia (AML) cell growth. Oncotarget. 2015; 7: 1395–407. 10.18632/oncotarget.6366 PMC4811468 26593251

[31] Thorburn A , Thamm DH , Gustafson DL . Autophagy and cancer therapy. Mol Pharmacol. 2014; 85: 830–8. 24574520 10.1124/mol.114.091850 PMC4014668

[32] Hong SK , Kim JH , Starenki D , *et al* Autophagy sensitivity of neuroendocrine lung tumor cells. Int J Oncol. 2013; 43: 2031–8. 24126619 10.3892/ijo.2013.2136 PMC3834067

[33] Wu YC , Wu WK , Li Y , *et al* Inhibition of macroautophagy by bafilomycin A1 lowers proliferation and induces apoptosis in colon cancer cells. Biochem Biophys Res Commun. 2009; 382: 451–6. 19289106 10.1016/j.bbrc.2009.03.051

[34] Kumar D , Shankar S , Srivastava RK . Rottlerin induces autophagy and apoptosis in prostate cancer stem cells *via* PI3K/Akt/mTOR signaling pathway. Cancer Lett. 2014; 343: 179–89. 24125861 10.1016/j.canlet.2013.10.003

[35] Heras‐Sandoval D , Perez‐Rojas JM , Hernandez‐Damian J , *et al* The role of PI3K/AKT/mTOR pathway in the modulation of autophagy and the clearance of protein aggregates in neurodegeneration. Cell Signal. 2014; 26: 2694–701. 25173700 10.1016/j.cellsig.2014.08.019

[36] Martelli AM , Evangelisti C , Chappell W , *et al* Targeting the translational apparatus to improve leukemia therapy: roles of the PI3K/PTEN/Akt/mTOR pathway. Leukemia. 2011; 25: 1064–79. 21436840 10.1038/leu.2011.46

[37] Sun B , Xu M . Matrine inhibits the migratory and invasive properties of nasopharyngeal carcinoma cells. Mol Med Rep. 2015; 11: 4158–64. 25633440 10.3892/mmr.2015.3276 PMC4394955

[38] Carew JS , Kelly KR , Nawrocki ST . Autophagy as a target for cancer therapy: new developments. Cancer Manag Res. 2012; 4: 357–65. 23091399 10.2147/CMAR.S26133 PMC3474143

