# Supplementary figures and images for: Matrine induces Akt/mTOR signalling inhibition‐mediated autophagy and apoptosis in acute myeloid leukaemia cells
Source: J Cell Mol Med. 2016 Dec 27;21(6):1171–81. doi: 10.1111/jcmm.13049 (PMC5431164; doi:10.1111/jcmm.13049)

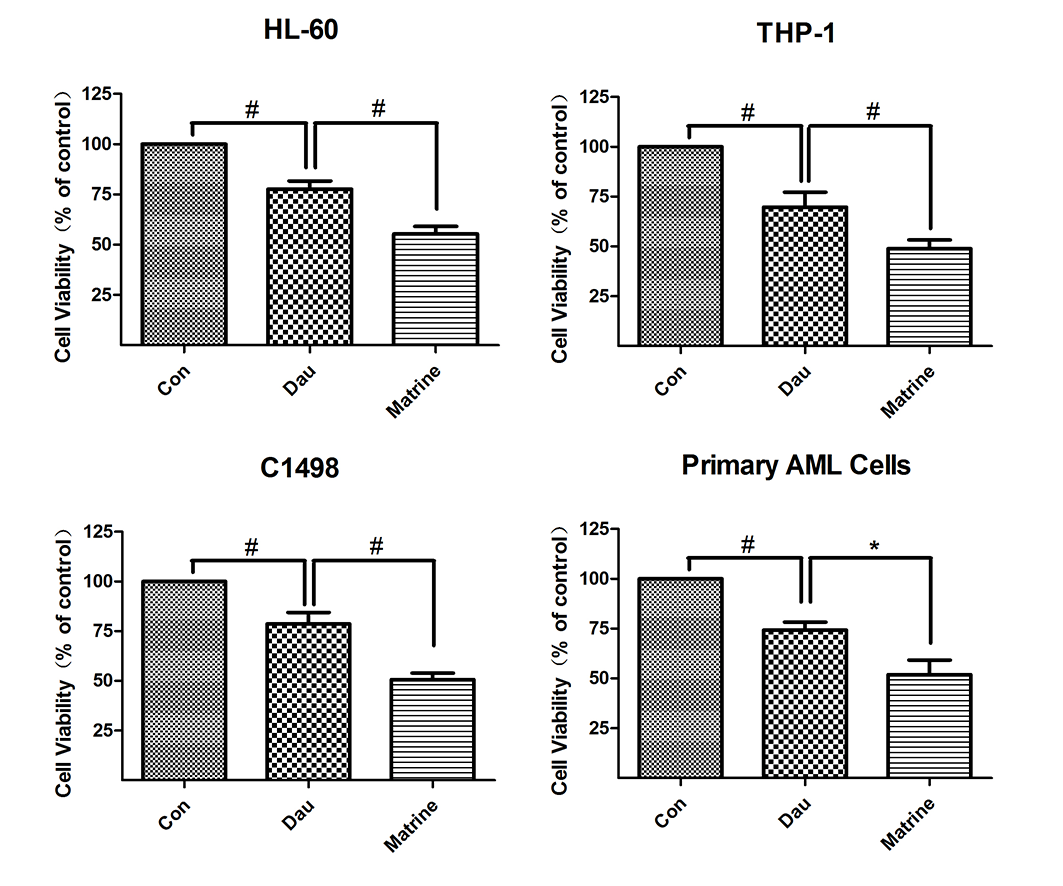

Supplement: Supplementary file 1 — Figure S1 After incubated with matrine (1.5 g/l) or daunorubicin (100 nM), the cell viability of AML cell lines HL‐60, THP‐1, C1498 and primary AML cells was measured by CCK‐8 assay. *P < 0.05, #P < 0.01, versus the respective control. [file JCMM-21-1171-s001.tif]

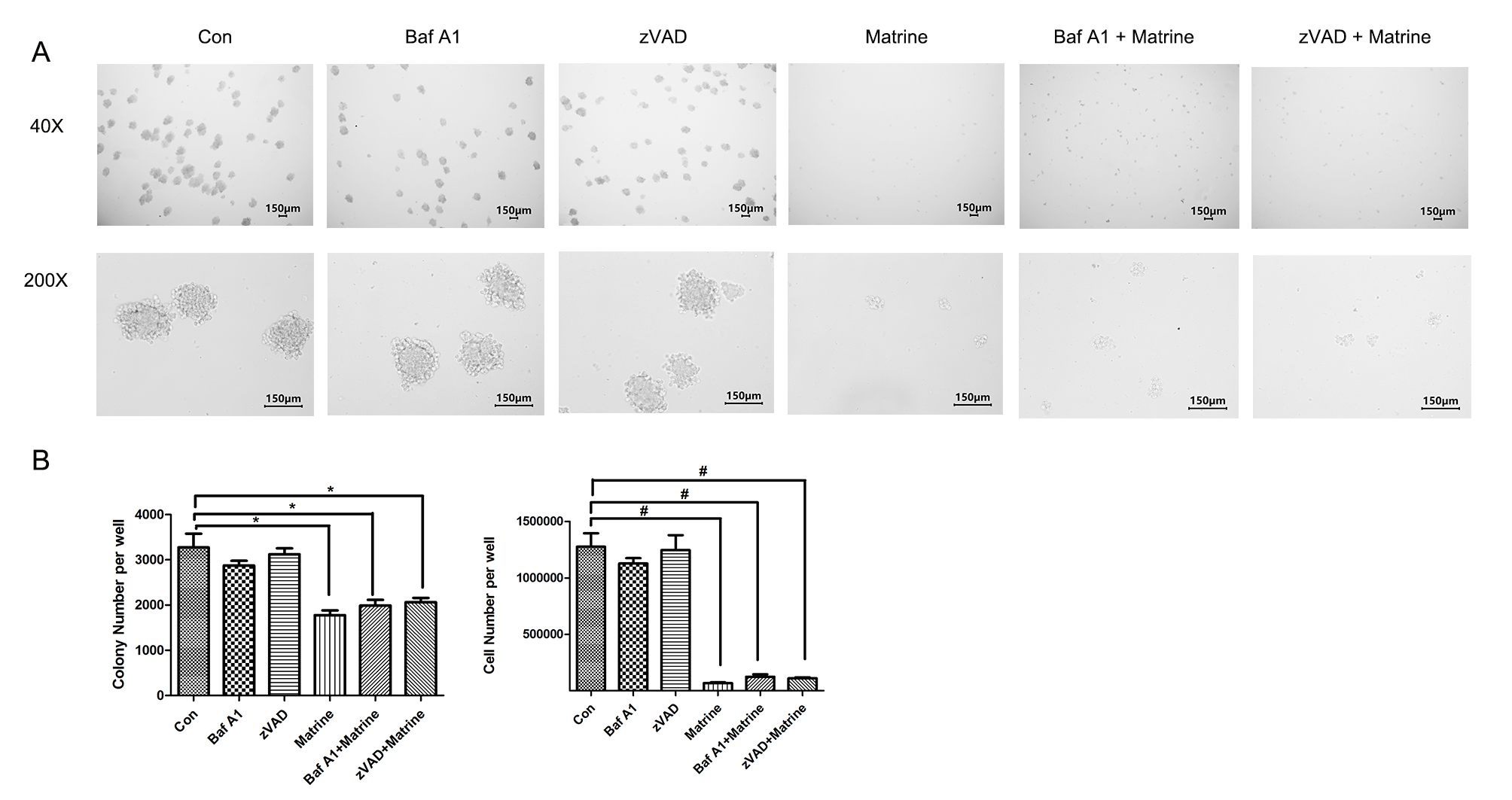

Supplement: Supplementary file 2 — Figure S2 (A) The morphological changes of colonies were observed under a microscopy in HL‐60 cells treated with 1.5 g/l matrine, 10 nM Baf A1, or 10 μM z‐VAD‐FMK for 8 days. Images shown were representatives of at least three independent experiments. (B) Quantitative data of colony and cell number were presented in bar charts. Results were expressed as mean ± S.E.M. representing at least three independent experiments. *P < 0.05, #P < 0.01, versus the respective control. [file JCMM-21-1171-s002.tif]

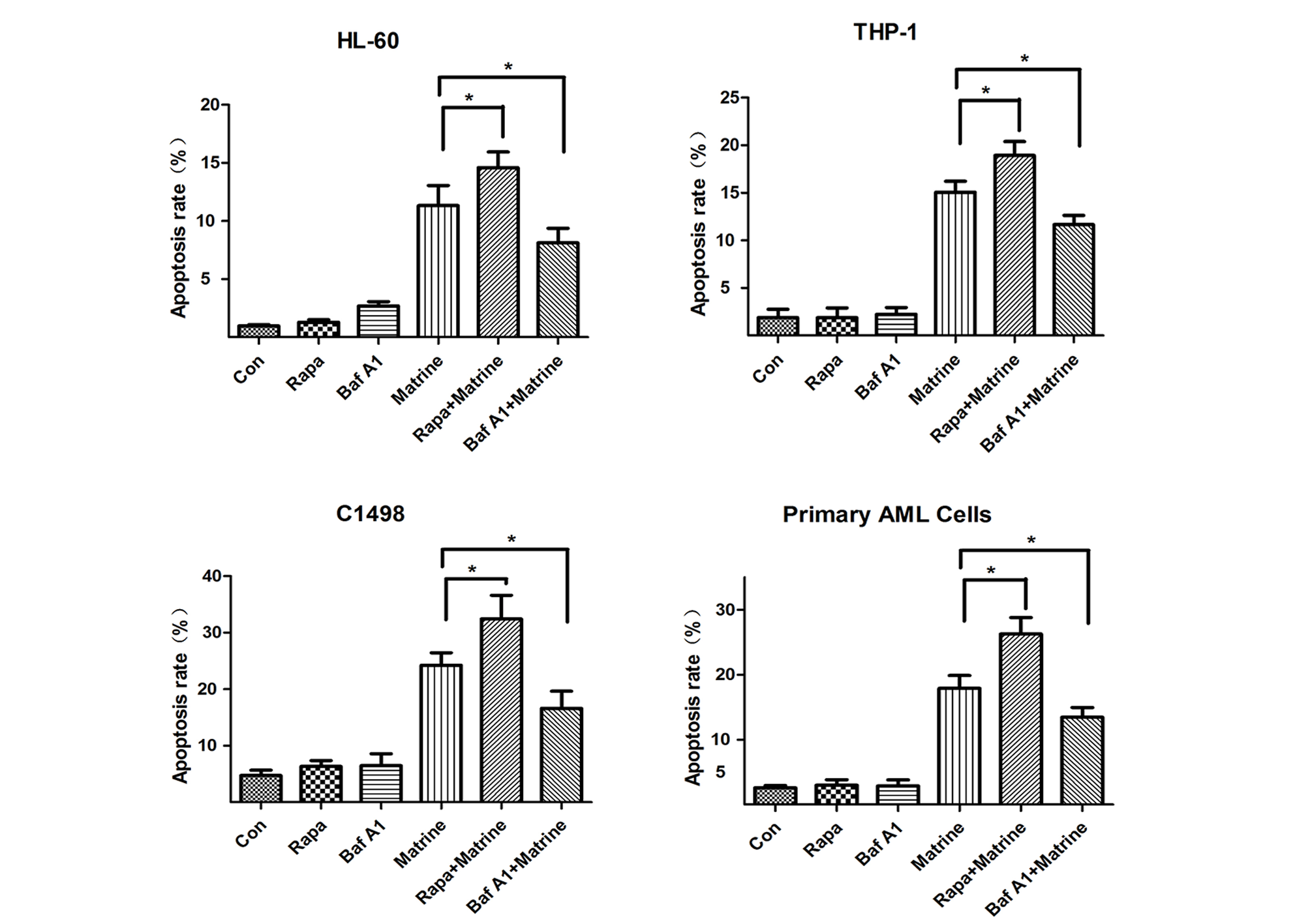

Supplement: Supplementary file 3 — Figure S3 AML cell lines HL‐60, THP‐1, and C1498 as well as primary AML cells were treated with 1.5 g/l matrine, either alone or in combination with 10 nM Baf A1 or 20 nM rapamycin (Rapa) for 24 hrs, and subsequently stained with Annexin V/PI for flow cytometry analysis. The percentage of apoptosis was presented in bar charts. Results were expressed as mean ± S.E.M. representing at least three independent experiments. *P < 0.05, versus matrine alone group. [file JCMM-21-1171-s003.tif]
