# Supplementary material for: Matrine induces Akt/mTOR signalling inhibition‐mediated autophagy and apoptosis in acute myeloid leukaemia cells
Source: J Cell Mol Med. 2016 Dec 27;21(6):1171–81. doi: 10.1111/jcmm.13049 (PMC5431164; doi:10.1111/jcmm.13049)
Supplement: Supplementary file 4 — Table S1 Characteristics of patients with acute myeloid leukemia. [file JCMM-21-1171-s004.docx]

Table S1. Characteristics of patients with acute myeloid leukemia

| Patient Sex Age Diagnosis |
| --- |

1 F 17 Acute myelomonocytic leukemia

2 M 28 Acute myelomonocytic leukemia

3 M 37 Acute myelomonocytic leukemia

4 F 40 Acute monoblastic leukemia

5 M 46 AML with myelodysplasia

6 F 40 AML with maturation

7 F 6 AML without maturation

8 M 48 Acute myelomonocytic leukemia

9 M 61 Acute monoblastic leukemia

10 M 19 Acute monoblastic leukemia

11 F 50 AML with maturation

12 M 24 AML with myelodysplasia

13 M 46 Acute myelomonocytic leukemia

14 F 36 AML with myelodysplasia

15 F 55 Acute myelomonocytic leukemia

16 M 64 AML with myelodysplasia

17 F 42 Acute monoblastic leukemia
